# Association study between genetic variants and the risk of schizophrenia in the Chinese population based on GWAS-implicated 6p21.3–23.1 human genome region: a case-control study

**DOI:** 10.1186/s12888-021-03496-5

**Published:** 2021-10-04

**Authors:** Gangqin Li, Jie Dai, Hao Liu, Yushan Lin, Qiaoni Liu, Kaiyuan Zheng, Suyu Li, Siyu Chen, Yi Ye

**Affiliations:** 1 Department of Forensic Psychiatry, West China School of Basic Medical Sciences & Forensic Medicine, Sichuan University, Chengdu, 610041 Sichuan China; 2 Department of Pathology, Sichuan Cancer Hospital, Chengdu, Sichuan China; 3 West China School of Basic Medical Sciences & Forensic Medicine, Sichuan University, Chengdu, Sichuan China; 4 Nanchong Psychosomatic Hospital, Nanchong, 637000 Sichuan China; 5 Department of Forensic Toxicological Analysis, West China School of Basic Medical Sciences & Forensic Medicine, Sichuan University, Chengdu, Sichuan China

**Keywords:** Schizophrenia, Single nucleotide polymorphism, Human leukocyte antigen, miR-219a-1

## Abstract

**Background:**

Schizophrenia is a polygenic disease; however, the specific risk genetic variants of schizophrenia are still largely unknown. Single nucleotide polymorphism (SNP) is important genetic factor for the susceptibility of schizophrenia. Investigating individual candidate gene contributing to disease risk remains important.

**Methods:**

In a case-control study, five SNPs located in 6p21.3-p23.1 including rs2021722 in human leukocyte antigen (HLA) locus and rs107822, rs383711, rs439205 and rs421446 within the upstream of microRNA-219a-1 were genotyped in 454 schizophrenia patients and 445 healthy controls to investigate the possible association between the loci and schizophrenia in a Han Chinese population.

**Results:**

Our results showed significant associations between the rs2021722 and schizophrenia in allele (A vs. G: adjusted OR = 1.661, 95%CI = 1.196–2.308), co-dominant (AG vs. GG: OR = 1.760, 95%CI = 1.234–2.510) and dominant genetic model (AG + AA vs. GG: OR = 1.756, 95%CI = 1.237–2.492), respectively. Haplotype analysis showed that TGGT and CAAC were protective factor for schizophrenia compared with TAAC haplotype (OR = 0.324, 95% CI = 0.157–0.672; OR = 0.423, 95% CI = 0.199–0.900).

**Conclusions:**

These findings indicate that rs2021722 in HLA locus might be involved in pathogenesis of schizophrenia and that genotypes AG and allele A of the locus are risk factors for schizophrenia in the Han Chinese population, confirming the association between immune system and schizophrenia.

## Background

Schizophrenia is a severe psychiatric disorder with a prevalence about 1 ~ 1.5% of the world population [1]. Interacting of multiple genetic, nutritional and environmental factors contributed to its etiology, while genetic factors provided basis for its susceptibility [2–4]. Although some investigations showed that genetic factors were the main causes of schizophrenia [5], the specific risk genetic variants of schizophrenia are still largely unknown. Recent genome-wide association studies (GWAS) indicated that schizophrenia was the result of hundreds or thousands of common genetic variants acting in concert to produce a neuropsychiatric phenotype [6]. Investigating individual candidate gene contributing to disease risks remains important. In 2011, the Schizophrenia Psychiatric Genome-Wide Association Study Consortium had reported the genome-wide association between the rs2021722 and schizophrenia in samples of European ancestry [7]. The rs2021722 is located within the human leukocyte antigen (HLA) locus on chromosome 6p21.3–23.1. This locus has been identified as one of the most robust genetic region susceptibility to schizophrenia based on European samples [8]. However, the association between the rs2021722 and schizophrenia has rarely been replicated in a large Han Chinese population. In this context, exploring the possible association between the rs2021722 and schizophrenia in Han Chinese population is in great need.

Although single nucleotide polymorphism (SNP) in coding genes plays an important role in the susceptibility to schizophrenia, it could not explain all of the genetic factors for schizophrenia. Therefore researchers began to focus on the effects of non-coding RNAs, especially microRNAs (miRNAs). miRNAs are small non-coding single stranded RNA molecules, which can regulate gene expression at the posttranscriptional level and in further, influence a great number of target genes and their functions. In recent years, accumulating evidence indicated that miRNAs may act as key regulators in a large number of different cellular processes, including neuronal proliferation and maturation, synaptogenesis and synaptic plasticity, dendrite and synaptic pruning [9, 10]. These processes play crucial roles in mental health. It is evident that miRNAs offer an exciting potential not only to understand the underlying mechanisms of schizophrenia, but also for the future development of antipsychotics [11–13] .

miR-219a-1, located in the schizophrenia susceptible region of 6p21.3, is an important regulator in N-methyl-D-aspartate receptors (NMDAR)-mediated glutamate signal pathway [14, 15]. This pathway is involved in synaptic plasticity and glutamatergic neurotransmission, and is regarded as the final common pathway on the road to schizophrenia [16]. Disruption of this signal pathway can lead to behavioral deficits, including schizophrenia [14]. Several lines of studies have suggested a link between miR-219a-1 dysregulation and NMDAR signaling in schizophrenia; however, the specific mechanism is still unknown. In this context, exploring the possible association between miR-219a-1 related SNPs and schizophrenia in Han Chinese population was the second aim of the present study.

In this study, we selected a GWAS identified schizophrenia-associated SNP rs2021722 in European population and aimed to investigate the possible association between the rs2021722 and schizophrenia in Han Chinese population. Considering the polygenetic nature of schizophrenia and the important role of miRNA for psychiatric disorders, we also selected SNPs within − 2 kb of miR-219a-1 to investigate whether these SNPs were individually or jointly associated with schizophrenia in Han Chinese population.

## Methods

### Participants

A case-control study was performed in 454 clinical diagnosed schizophrenia patients (312 males, mean age = 48.35 ± 12.36 years; 142 females: mean age = 47.20 ± 12.10 years) and 445 healthy checking-up individuals (327males, mean age = 46.53 ± 12.42 years; 118 females: mean age = 47.83 ± 12.74 years) from January of 2019 to October of 2020 in Sichuan Province of China.

Schizophrenia patients were diagnosed by at least two psychiatrists according to the International Statistical Classification of Diseases and Related Health Problems, Tenth Revision (ICD-10) criteria. The controls were healthy checking-up individuals without any personal or family history of neuropsychiatric disorder or any abuse of addictive drug. All included participants were Han Chinese descendants. Peripheral blood sample (2 ml) of each participant was collected in sterile tubes with EDTA–Na2 anticoagulants and stored at − 20 °C.

This study was approved by the Medical Ethics Committee of Sichuan University (reference number: K2018092) and written informed consents were obtained from all participants.

### SNPs selection and genotyping

The rs2021722 was a GWAS identified schizophrenia-related SNP in European samples, which is of great value to be replicated in Han Chinese population. Except for the rs202121722, we also selected SNPs within 2 kb upstream of miR-219a-1, which is located in the schizophrenia susceptible region of 6p21.3–23.1. The criteria for SNPs selection were that minor allele frequency is more than 10% in Han Chinese population. Finally, four SNPs (i.e., rs107822, rs383711, rs439205 and rs421446) were enrolled in this study.

Genomic DNA was extracted from the peripheral blood using Tiangen human blood genome isolation Kit (Tiangen, Beijing, China) according to the manufacturer’s protocol. Genotypes of rs107822, rs383711and rs439205 were detected by TaqMan genotyping discrimination assay using ABI7500 PCR system (Applied Biosystems, Foster City, USA). Genotypes of rs2021722 and rs421446 were detected by polymerase chain reaction-restriction fragment length polymorphism (PCR-RFLP) assay. The primers of the rs2021722 were 5′-gtggggatttctagcgttca-3′ (forward) and 5′-cccagtgactgtggatgatg-3′(reverse), which generated a 135-bp fragment. The fragment was then digested by AluI (New England BioLabs), and may produce three genotypes as GG (135 bp), AG (135, 76 and 59 bp), and AA(76 and 59 bp). The primers for the rs421446 were 5′-gccagactggacaccaagat-3′(forward) and 5′- gtgacactccgggtcttctt-3′ (reverse), which generated a 150-bp fragment. The fragment was then digested by Hpy888-III (New England BioLabs), and may produce three genotypes as CC (150 bp), CT (150, 130 and 20 bp), and TT(130 and 20 bp). Restriction fragments were distinguished on 8% polyacrylamide gel and visualized by silver staining to determine the genotypes. Negative control was used in each experiment and Sanger sequencing was done to confirm the genotyping result.

### Statistical analysis

Genotype frequencies and Hardy–Weinberg equilibrium were calculated by SNPStats. Independent sample *t*-test was performed to compare the difference in quantitative data, while chi-square test was used to examine distribution difference of allele or genotype in two groups. Odds ratios (ORs) and 95% confidence intervals (95% CIs) were selected to estimate the strength between the five loci and risk of schizophrenia. All statistics were performed using SPSS software 17.0 (SPSS Inc., Chicago, IL, USA) and *p* < 0.05 was considered as statistical significance.

## Results

The genotype distributions of the five SNPs in the control group met the requirements of the Hardy-Weinberg equilibrium. Demographic information of the participants is present in Table 1. There were no significant differences in age and gender distributions between patients and controls.

**Table 1:** Demographic characteristics of the included schizophrenia patients and healthy controls

	HC^a^			SCZ^b^			*p*
	Total	Male	Female	Total	Male	Female	
Number	445	327	118	454	312	142	0.115
Age (M^c^ ± SD^d^)	46.88 (±12.50)	46.53 (± 12.42)	47.83 (± 12.74)	47.99 (±12.28)	48.35 (± 12.36)	47.20 (±12.10)	0.179

Notes: ^a^HC = healthy controls; ^b^SCZ = schizophrenia patients; M^c^ = Mean; SD^d^ = Standard Deviation

### The genotype and allele distributions

The genotype and allele distributions of the five SNPs are present in Table 2. Among the five SNPs, only rs2021722 showed significant differences in allele and genotype frequencies between cases and controls. Frequencies of allele A (OR = 1.661, 95% CI = 1.196–2.308) and genotype AG (OR = 1.760, 95%CI = 1.234–2.510) at the rs2021722 were higher in patient group than that in control group, suggesting that individuals carrying allele A could increase the risk of developing schizophrenia. No differences were observed in other four SNPs between cases and controls.

**Table 2:** The distribution of alleles and genotypes for 5 SNPs between schizophrenia patients and healthy control subjects

	SNP	HCs^a^, N^b^(%)	SCZs^c^, N(%)	OR^d^ (95% CI^e^)	*p*
**rs107822**					
	T	569 (63.9)	564 (62.1)	1.000(ref)	
	C	321 (36.1)/(39.6)^f^	344 (37.9)	1.081 (0.893–1.309)	0.425
codominant	TT	191 (42.9)	185 (40.8)	1.000(ref)	
	CC	67 (15.1)	75 (16.5)	1.156 (0.785–1.701)	0.463
	CT	187 (42.0)	194 (42.7)	1.071 (0.805–1.424)	0.637
dominant	TT	191 (42.9)	185 (40.8)	1.000(ref)	
	CC/CT	254 (57.1)	269 (59.2)	1.093 (0.839–1.425)	0.509
recessive	TT- CT	378 (84.9)	379 (83.5)	1.000(ref)	
	CC	67 (15.1)	75 (16.5)	1.116 (0.780–1.599)	0.547
**rs383711**					
	A	454 (51.0)	451 (49.7)	1.000(ref)	0.569
	G	436 (49.0)/(49.2)^f^	457 (50.3)	1.055 (0.877–1.269)	
codominant	AA	114 (25.6)	112 (24.7)	1.000(ref)	
	AG	226 (50.8)	227 (50.0)	1.022 (0.743–1.407)	0.892
	GG	105 (23.6)	115 (25.3)	1.115 (0.769–1.616)	0.566
dominant	AA	114 (25.6)	112 (24.7)	1.000(ref)	
	AG- GG	331 (74.4)	342 (75.3)	1.052 (0.778–1.422)	0.743
recessive	AA- AG	340 (76.4)	339 (74.7)	1.000(ref)	
	GG	105 (23.6)	115 (25.3)	1.098 (0.810–1.489)	0.545
**rs439205**					
	A	549 (61.7)	560 (61.7)	1.0000(ref)	
	G	341 (38.3)/(39.6)^f^	348 (38.2)	1.000 (0.827–1.210)	0.996
codominant	AA	174 (39.1)	181 (39.9)	1.000	
	AG	201 (45.2)	198 (43.6)	0.947 (0.711–1.261)	0.709
	GG	70 (15.7)	75 (16.5)	1.030 (0.0.700–1.516)	0.881
dominant	AA	174 (39.1)	181 (39.9)	1.000(ref)	
	AG- GG	271 (60.9)	273 (60.1)	0.968 (0.741–1.265)	0.814
recessive	AA- AG	375 (84.3)	379 (83.5)	1.000(ref)	0.748
	GG	70 (15.7)	75 (16.5)	1.060 (0.743–1.513)	
**rs2021722**					
	G	827 (92.9)	806 (88.8)	1.000(ref)	
	A	63 (7.1)/(10.9)^f^	102 (11.2)	1.661 (1.196–2.308)	0.002
codominant	GG	384 (86.3)	355 (78.2)	1.000(ref)	
	AA	2 (0.4)	3 (0.7)	1.623 (0.270–9.767)	0.933
	AG	59 (13.3)	96 (21.1)	1.760 (1.234–2.510)	0.002
dominant	GG	384 (86.3)	355 (78.2)	1.000(ref)	
	AG-AA	61 (13.7)	99 (21.8)	1.756 (1.237–2.492)	0.002
recessive	GG-AG	443 (99.5)	451 (99.3)	1.000(ref)	0.670
	AA	2 (0.4)	3 (0.7)	1.473 (0.245–8.860)	
**rs421446**					
	G	598 (67.2)	607 (66.8)	1.000(ref)	
	A	292 (32.8)/(38.3)^f^	301 (33.1)	1.016 (0.834–1.236)	0.878
codominant	GG	196 (44.0)	204 (44.9)	1.000(ref)	
	AG	206 (46.3)	199 (43.8)	0.928 (0.704–1.224)	0.597
	AA	43 (9.7)	51 (11.2)	1.140 (0.726–1.788)	0.570
dominant	GG	196 (44.0)	204 (44.9)	1.000(ref)	
	AG- AA	249 (56.0)	250 (55.1)	0.965 (0.741–1.255)	0.789
recessive	GG- AG	402 (90.3)	403 (88.8)	1.000(ref)	
	AA	43 (9.7)	51 (11.2)	1.183 (0.771–1.816)	0.442

Notes: ^a^*HC* Healthy controls, ^b^*N* number of the group, ^c^*SCZ* Schizophrenia patient, ^d^*OR* Odds ratio, ^e^*CI* Confidence interval, ^f^ = the number in the bracket behind the slash is minor allele frequency in East Asian sample (EAS), data are based on NCBI SNPdatabase (http://www.ncbi.nlm.nih.gov/snp/; accessed on 10 June 2021)

### Combined effects of the five SNPs

We also examined the combined effects of rs2021722 and other four SNPs on schizophrenia risk. As shown in Table 3, the carriers with the combined genotypes of rs2021722 AA/AG + rs107822 TT (OR = 1.828, 95% CI = 1.096–3.048), rs2021722 AA/AG + rs107822 CC/CT (OR = 1.957, 95% CI = 1.188–3.225), rs2021722 AA/AG + rs383711 GG/AG (OR = 1.813, 95% CI = 1.141–2.883), rs2021722 AA/AG + rs439205 GG/AG (OR = 1.773, 95%CI = 1.093–2.876), rs2021722 AA/AG + rs421446 GG (OR = 1.703, 95%CI = 1.027–2.823) and rs2021722 AA/AG + rs421446 AA/AG (OR = 1.751, 95% CI = 1.065–2.878) had significant increased risk of schizophrenia.

**Table 3:** The combined genotypes frequencies of rs2021722 with other four SNPs in two groups

Polymorphisms	HCs, N (%)	SCZs, N (%)	OR (95% CI)	*p*
**rs2021722 & rs107822**				
rs2021722 GG + rs107822 TT	161 (36.2)	138 (30.4)	1.000(ref)	
rs2021722 GG + rs107822 CC/CT	223 (50.1)	217 (47.8)	1.135 (0.846–1.524)	0.398
rs2021722 AA/AG + rs107822 TT	30 (6.7)	47 (10.4)	1.828 (1.096–3.048)	0.020
rs2021722 AA/AG + rs107822 CC/CT	31 (7.0)	52 (11.5)	1.957 (1.188–3.225)	0.008
**rs2021722 & rs383711**				
rs2021722 GG + rs383711AA	100 (22.5)	88 (19.4)	1.000(ref)	
rs2021722 GG + rs383711 GG/AG	284 (63.8)	267 (58.8)	1.068 (0.767–1.488)	0.696
rs2021722 AA/AG + rs383711 AA	14 (3.1)	24 (5.3)	1.948 (0.949–3.997)	0.066
rs2021722 AA/AG + rs383711 GG/AG	47 (10.6)	75 (16.5)	1.813 (1.141–2.883)	0.012
**rs2021722 & rs439205**				
rs2021722 GG + rs439205 AA	147 (33.0)	139 (30.6)	1.000(ref)	
rs2021722 GG + rs439205 GG/AG	237 (53.3)	216 (47.6)	0.964 (0.717–1.296)	0.808
rs2021722 AA/AG + rs439205 AA	27 (6.1)	42 (9.3)	1.645 (0.962–2.812)	0.067
rs2021722 AA/AG + rs439205GG/AG	34 (7.6)	57 (12.6)	1.773 (1.093–2.876)	0.020
**rs2021722 & rs421446**				
rs2021722 GG + rs421446 GG	166 (37.3)	156 (34.4)	1.000(ref)	
rs2021722 GG + rs421446 AA/AG	218 (49.0)	199 (43.8)	0.971 (0.726–1.299)	0.845
rs2021722 AA/AG + rs421446 GG	30 (6.7)	48 (10.6)	1.703 (1.027–2.823)	0.038
rs2021722 AA/AG + rs421446 AA/AG	31 (7.0)	51 (11.2)	1.751 (1.065–2.878)	0.026

Notes: ^a^*HC* Healthy controls, ^b^*N* number of the group, ^c^*SCZ* Schizophrenia patient, ^d^*OR* Odds ratio, ^e^*CI* Confidence interval

### Linkage disequilibrium test and haplotype analysis of 4 SNPs within miR-219a-1

The four SNPs (rs107822, rs383711, rs439205 and rs421446) located within 2 kb upstream of miR-219a-1 had strong linkage disequilibrium with each other (D′ = 0.832–0.909, details are present in Table 4). We identified three protective haplotype blocks according to the pairwise linkage disequilibrium analysis (Table 5). Compared to TAAC, TGGT and CAAC acted as a risk haplotype (OR = 0.324, 95% CI = 0.157–0.672; OR = 0.423, 95% CI = 0.199–0.900) for schizophrenia.

**Table 4:** Linkage disequilibrium tests of four SNPs within miR-219a-1

	rs107822, D′(r^2^)	rs383711, D′(r)	rs439205, D′(r)	rs421446, D′(r)
rs107822	–	0.895 (0.439)	0.849 (0.681)	0.832 (0.580)
rs383711	0.895 (0.439)	–	0.887 (0.495)	0.909 (0.412)
rs439205	0.849 (0.681)	0.887 (0.495)	–	0.884 (0.619)
rs421446	0.832 (0.580)	0.909 (0.412)	0.884 (0.619)	–

**Table 5:** Haplotype analysis of four SNPs within miR-219a-1 between two groups

	HCs, N(%)	SCZs, N(%)	OR (95% CI)	*p*
TAAC	402 (45.2)	413 (45.5)	1.000(ref)	–
CGGT	237 (26.6)	269 (29.6)	1.105 (0.885–1.380)	0.379
TGAC	96 (10.8)	115 (12.7)	1.166 (0.861–1.580)	0.321
CGGC	38 (4.3)	50 (5.5)	1.281 (0.822–1.996)	0.273
TGGT	30 (3.4)	10 (1.1)	0.324 (0.157–0.672)	0.002
CAAC	23 (2.6)	10 (1.1)	0.423 (0.199–0.900)	0.022
TAGC	18 (2.0)	10 (1.1)	0.541 (0.247–1.186)	0.120
TAAT	6 (0.7)	10 (1.1)	1.622 (0.584–4.505)	0.349
CGAT	12 (1.3)	5 (0.5)	0.406 (0.142–1.162)	0.083
TGGC	12 (1.4)	5 (0.5)	0.406 (0.142–1.162)	0.083

Notes: ^a^*HC* Healthy controls, ^b^*N* number of the group, ^c^*SCZ* Schizophrenia patient, ^d^*OR* Odds ratio, ^e^*CI* Confidence interval

### Expression quantitative trait loci (eQTL) analysis of the 4 SNPs within miR-219a-1

**Fig. 1:**
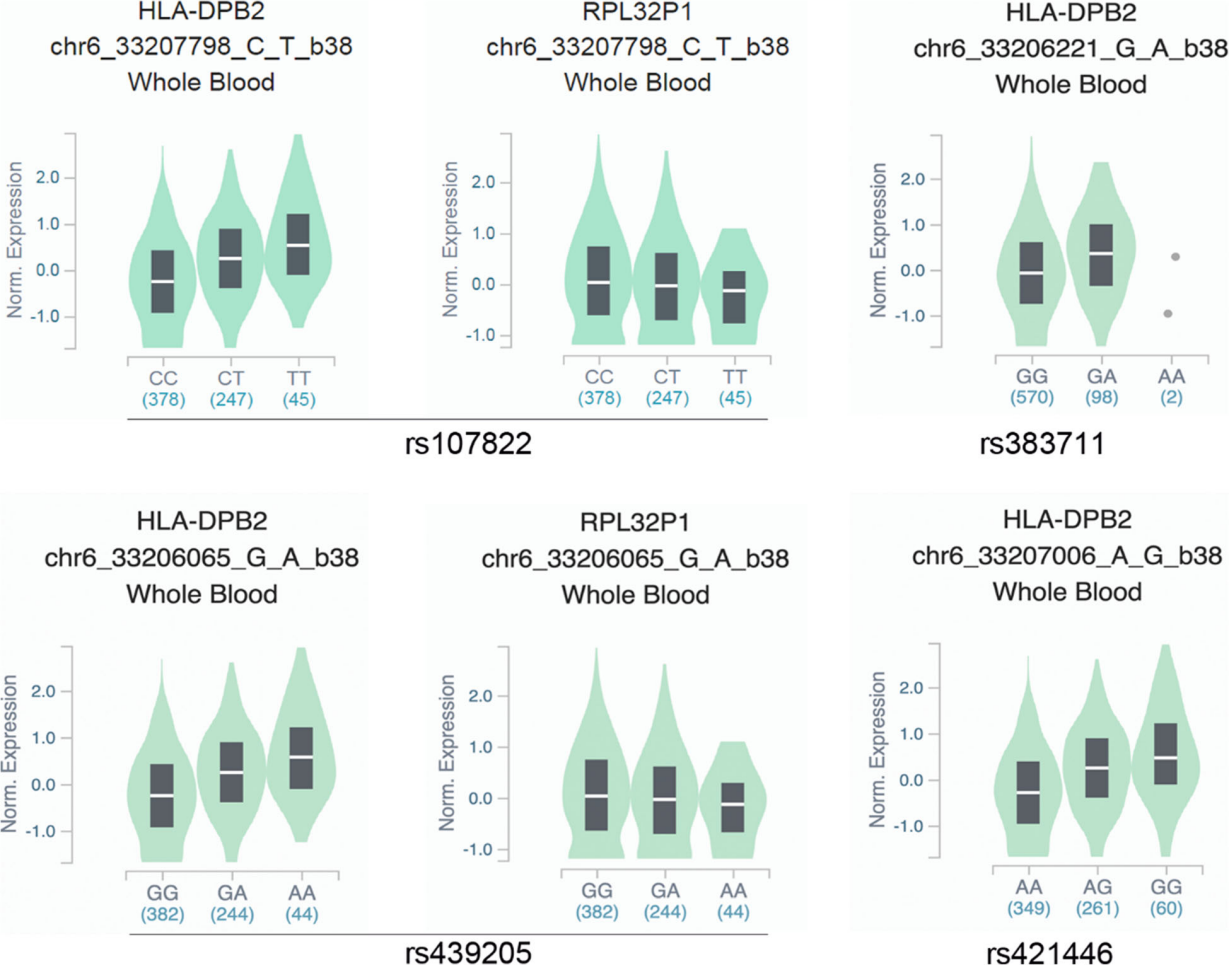
Genes associated with the 4 SNPs within miR-219a-1 in whole blood. Note: Data are from the https://www.gtexportal.org/home/, accessed on 10 June 2021

Expression Quantitative trait loci (eQTL) analysis showed that all the four SNPs within miR-219a-1 are associated with HLA-DPB2 and would significantly influence the expression of HLA-DPB2(all *p* < 0.001) in whole blood (see details in Fig. 1).

## Discussion

In this case-control study, we investigated the association between five SNPs on the chrome 6 (6p21–22) and the risk of schizophrenia in a Han Chinese population. In consistent with the GWAS findings in European samples [7], we found that the rs2021722 in HLA locus was also associated with the risk of schizophrenia in our Han Chinese population. Subjects carrying rs2021722 AG genotype and A allele had increased risk of schizophrenia.

The rs2021722 is located within the HLA locus that was identified as one of the most significant determinant of schizophrenia susceptibility in GWAS [17, 18]. Emerging evidence has indicated that immune system and central nervous system (CNS) may have common evolutionary origin and interact with each other in many pathways [19, 20]. Schizophrenia has been extensively studied to be abnormal in immune systems [21], for example, several meta-analysis have reported that immune system was over active in patients with medication-naive first episode psychosis [22–24]. Immune dysfunctions may affect neurobiological circuits including changed neurotransmitter metabolisms contributing to pathophysiological alterations in schizophrenia [23]. Another line of evidence suggested the imbalance between type-1 and type-2 immune responses of schizophrenia might lead to the glutamatergic-dopaminergic dysregulation and then contribute to the clinical symptoms of psychosis [22].

Except the immunological function, increasing evidence has shown that HLA proteins have fundamental non-immune effect in neurogenesis, neuronal differentiation and migration, synaptogenesis, synaptic plasticity and pruning [25–27]. These processes have all been involved in the pathogenesis of schizophrenia [28, 29]. CNS structure integrity(e.g. enlarged ventricles) was one of the frequently reported structural abnormalities in schizophrenic patients and animal models [30, 31]. Tao et al. reported that the rs2021722 was significantly associated with cortical thickness in antipsychotic-naïve schizophrenia patients, indicating that the immune system may play critical roles in the pathology of schizophrenia via the modulation of the development of cerebral cortical structures [32]. Together, although the specific mechanism of interaction between immune system and schizophrenia is not fully known, the present study confirmed the association between the rs2021722 in the HLA region and the risk of schizophrenia in Han Chinese population. Mechanisms on how genetic variability like rs2021722 in HLA region leads to schizophrenia are worthy of further investigation.

miR-219a-1, located in the schizophrenia susceptible region of 6p21.3, has important role in the structure and function of CNS. It could inhibit NMDA signaling by attenuating NMDA-induced neuronal depolarization and is an important regulator in NMDA receptors-mediated glutamate signal pathway [33]. The glutamate pathway is involved in synaptic plasticity and glutamatergic neurotransmission, and was regarded as the final common pathway on the road to schizophrenia [16]. Except the effect on NMDA signal pathway, miR-219a-1 was also reported to positively regulate oligodendrocyte differentiation, myelin and maturation [34, 35], and play a role in circadian rhythm [33]. These processes mentioned above are all involved with pathogenesis of schizophrenia, indicating that the association between miR-219a-1 and schizophrenia. Four SNPs (rs107822, rs383711, rs439205 and rs421446) within 2 kb upstream of miR-219a-1 were selected in the current study to explore their associations with schizophrenia. eQTL analysis showed that all the four SNPs within miR-219a-1 could significantly influence the expression of HLA-DPB2. HLA-DPB2 is a pseudogene located in HLA region, which is strongly relevant to immune-related biological functions (e.g. adaptive immune response) by influencing the expression and activation of immune cells such as monocytes, NK cell and T cell [36]. In light of the association between immune system and schizophrenia, it might speculate that these four SNPs have some potential association with the pathogenesis of schizophrenia. Sun et al. had reported that the rs107822 within miR-219a-1 was significantly associated with the risk of schizophrenia and genotypes TC/CC and allele C of the locus may be used as predictive factors for the etiology of schizophrenia [37]. Zhang et al. also found that rs107822 within miR-219a-1 was nominally associated with schizhophrenia [15]. However, we failed to find any difference of the four SNPs within 2kp upstream of miR-219a-1 between schizophrenia patients and healthy controls in the current study. Samples from different geographical area and relatively small sample size may account for the differences due to heterogeneity [38]. Further studies with larger sample size are required to investigate the association between miR-219 and related SNPs and schizophrenia.

In order to investigate the interaction effect of the rs2021722 and other four SNPs on schizophrenia, combined analysis was performed in our study. The results showed that there was significantly combined effect of the rs2021722 and other four SNPs within miR-219a-1 on the risk of schizophrenia. Subjects carrying genotypes of rs2021722 AA/AG combined with any other four SNPs (rs107822 TT, rs107822 CC/CT, rs383711 GG/AG, rs439205GG/AG, rs421446 GG and rs421446 AA/AG) had an increased risk of schizophrenia. Smrt et al. had reported that miR-137 regulated neuronal maturation by targeting ubiquitin ligase Mind Bomb-1 [39]. The rs2021722 is an intron variant in Tripartite motif (TRIM), and most of the TRIM family proteins have E3 ubiquitin ligase activities with various functions including apoptosis, autophagy, innate immunity, and carcinogenesis [40]. This finding suggests that miR-137 may interact with the rs2021722 and jointly contribute to schizophrenia. It is worthy of further study to test whether there is corporation between miR-219a-1 and the rs2021722 in TRIM 26 for the pathogenesis of schizophrenia.

In the end, there still have some limitations in our study. Firstly, the sample size was relatively small, and only Han Chinese population was included. Further studies with larger sample size and multiple ethnic populations are needed to replicate our findings. Secondly, the current study had only five SNPs included, future studies could enroll additional potential functional SNPs affecting the expression of HLA and miR-219 to investigate the potential causal effects on schizophrenia under the framework of Mendelian Randomization analysis [41–43], and we may construct machine learning models to assist early diagnosis of schizophrenia based on these associated genetic variants [44, 45].

In conclusion, our study showed that the rs2021722 was associated with susceptibility to schizophrenia in Han Chinese population and genotype AG and allele A in this locus may be used as risk genetic markers for the disorder. Additionally, the results also indicated the potential interaction effects of the rs2021722 and other four SNPs within miR-219a-1 on the risk of schizophrenia. The specific mechanisms of these five SNPs on schizophrenia are worthy of future investigation. These findings might provide tools for better diagnostic and therapeutic characterization of schizophrenia.

## Data Availability

The data-sets used and/or analyzed during the current study available from the corresponding author on reasonable request.

## Abbreviations

SNP: Single nucleotide polymorphism
miRNA: Micro ribonucleic acid
GWAS: Genome-wide association study
HLA: Human leukocyte antigen
TRIM: Tripartite motif
CNS: Central nervous system
NMDAR: N-methyl-D-aspartate receptors
ICD-10: International Statistical Classification of Diseases and Related Health Problems-Tenth Revision
HC: Healthy controls
SCZ: Schizophrenia patient
OR: Odds ratio
CI: Confidence interval
SD: Standard deviation
